# Evaluation of Artificial Intelligence Methods to Estimate the Compressive Strength of Geopolymers

**DOI:** 10.3390/gels8050271

**Published:** 2022-04-26

**Authors:** Yong Zou, Chao Zheng, Abdullah Mossa Alzahrani, Waqas Ahmad, Ayaz Ahmad, Abdeliazim Mustafa Mohamed, Rana Khallaf, Samia Elattar

**Affiliations:** 1 School of Civil Engineering, Wuhan University, Wuhan 430072, China; 2 Department of Civil and Environmental Engineering, University of Texas at San Antonio, San Antonio, TX 78249, USA; 13808698522@163.com; 3 Department of Civil Engineering, College of Engineering, Taif University, P.O. Box 11099, Taif 21944, Saudi Arabia; amyalzahrani@tu.edu.sa; 4 Department of Civil Engineering, COMSATS University Islamabad, Abbottabad 22060, Pakistan; a.ahmad8@nuigalway.ie; 5 MaREI Centre, Ryan Institute and School of Engineering, College of Science and Engineering, National University of Ireland Galway, H91 HX31 Galway, Ireland; 6 Department of Civil Engineering, College of Engineering in Al-Kharj, Prince Sattam bin Abdulaziz University, Al-Kharj 11942, Saudi Arabia; a.bilal@psau.edu.sa; 7 Building & Construction Technology Department, Bayan College of Science and Technology, Khartoum 210, Sudan; 8 Structural Engineering and Construction Management Department, Faculty of Engineering and Technology, Future University in Egypt, New Cairo 11845, Egypt; rana.khallaf@fue.edu.eg; 9 Department of Industrial & Systems Engineering, College of Engineering, Princess Nourah bint Abdulrahman University, P.O. Box 84428, Riyadh 11671, Saudi Arabia; saelattar@pnu.edu.sa

**Keywords:** geopolymers, concrete, modeling, predictions, compressive strength

## Abstract

The depletion of natural resources and greenhouse gas emissions related to the manufacture and use of ordinary Portland cement (OPC) pose serious concerns to the environment and human life. The present research focuses on using alternative binders to replace OPC. Geopolymer might be the best option because it requires waste materials enriched in aluminosilicate for its production. The research on geopolymer concrete (GPC) is growing rapidly. However, substantial effort and expenses are required to cast specimens, cures, and tests. Applying novel techniques for the said purpose is the key requirement for rapid and cost-effective research. In this research, supervised machine learning (SML) techniques, including two individual (decision tree (DT) and gene expression programming (GEP)) and two ensembled (bagging regressor (BR) and random forest (RF)) algorithms were employed to estimate the compressive strength (CS) of GPC. The validity and comparison of all the models were made using the coefficient of determination (R^2^), k-fold, and statistical assessments. It was noticed that the ensembled SML techniques performed better than the individual SML techniques in forecasting the CS of GPC. However, individual SML model results were also in the reasonable range. The R^2^ value for BR, RF, GEP, and DT models was 0.96, 0.95, 0.93, and 0.88, respectively. The models’ lower error values such as mean absolute error (MAE) and root mean square errors (RMSE) also verified the higher precision of ensemble SML methods. The RF (MAE = 2.585 MPa, RMSE = 3.702 MPa) and BR (MAE = 2.044 MPa, RMSE = 3.180) results are better than the DT (MAE = 4.136 MPa, RMSE = 6.256 MPa) and GEP (MAE = 3.102 MPa, RMSE = 4.049 MPa). The application of SML techniques will benefit the construction sector with fast and cost-effective methods for estimating the properties of materials.

## 1. Introduction

Cement concrete is the most extensively used building material worldwide [1,2,3,4]. Cement concrete is composed primarily of different types of aggregates, water, and binding materials such as ordinary Portland cement (OPC) [5,6,7]. OPC is considered the third-most extremely energy-demanding substance on the planet after aluminum and steel, accounting for 7% of the total energy required by the industry [8,9,10]. Regrettably, the production of OPC generates vast amounts of greenhouse gas discharges such as CO_2_, which contribute substantially to climate change [11,12]. It is predicted that the manufacture of OPC produces 1.35 billion tons of greenhouse gas discharges per annum [13]. Therefore, researchers have centered their efforts on reducing OPC utilization due to the introduction of alternative binders. Alkali-activated materials, such as geopolymers, may be preferable to CBCC [14,15,16,17]. Alkali-activated materials are made when precursors and activators react. These were split into two groups according to the calcium level of the objects created during the chemical reaction: these are rich in calcium, having a Ca/(Si + Al) ratio of more than one, and others that have low calcium levels are called geopolymers [18].

**Figure 1:**
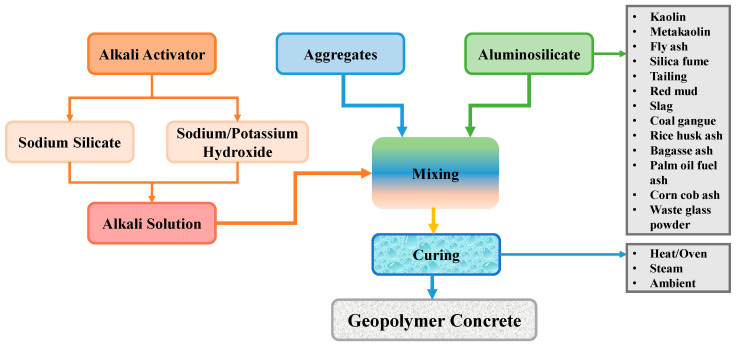
Schematic representation of the geopolymer production process [10].

**Figure 2:**
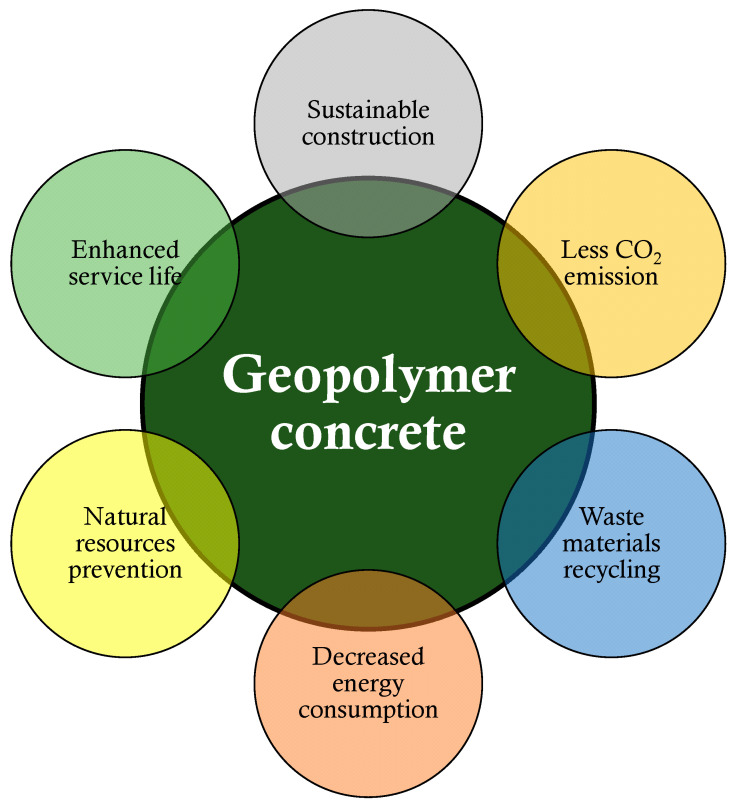
Benefits of geopolymer concrete incorporating waste materials.

A geopolymer is actually a type of new binder that was created to be utilized in the concrete manufacturing process in place of OPC [19,20,21,22]. The purpose is to create an OPC-free, environmentally friendly, and sustainable building material. As industry and population growth continue to expand, a substantial number of various types of waste materials are created and disposed of in landfills. These waste materials include rice husk ash, waste glass powder, ground granulated glass furnace slag, silica fume and fly ash, etc. Because these waste materials are pollution sources, dumping them in landfills is hazardous to the ecosystem [23,24,25,26,27,28]. As geopolymer concrete (GPC) demands raw elements with more aluminosilicate levels and are available in the waste materials, recycling this type of materials to manufacture GPC will decrease environmental pollution [29,30,31]. The process of GPC production has been depicted in Figure 1, indicating the various types of ingredients and curing regimes used to produce GPC. The utilization of these types of waste materials will benefit both the environmental conditions and the economy, as shown in Figure 2, because these wastes are plentiful, and the necessity for inexpensive housing will grow as the population grows [32,33]. Globally, GPC is gaining popularity in the field of research, and it may finally become the best green building material [34,35,36]. Nevertheless, GPC has the potential to significantly contribute to the long-term survival of both CBCC technology and the building industry.

Contemporary innovations in artificial intelligence (AI) have explained the wide application of supervised machine learning (SML) approaches for forecasting the characteristics of several materials [37,38,39,40,41,42,43]. Ahmad et al. [14] performed a comparative study on various SML approaches, i.e., decisions tree (DT), AdaBoost, and bagging regressor (BR), to forecast the compressive strength (CS) of GPC incorporating fly ash. It was noticed that the BR model had the utmost precision compared to the other models studied. In another study by Ahmad et al. [44], the CS of concrete incorporating recycled aggregates was anticipated using ANN and GEP. It was described that the GEP technique was more precise in terms of anticipating the required result as opposed to the ANN ML technique. Song et al.’s [45] research employed an ANN technique to investigate the CS of concrete having waste material and successfully forecasted the required outcome. The study also reported that these ML approaches could be successfully employed to forecast any type of mechanical property of concrete. Nguyen et al. [46] used a variety of SML techniques to anticipate tensile strength along with the CS of high-performance concrete. They concluded that the ensembled SML techniques were more precise than the individual SML techniques. This is because the individual ML algorithms normally make use of weak learners such as DT and MLP to generate a better model. Thus, various scholars reported distinct SML techniques with higher accuracies for the estimation of material properties. Therefore, it is vital to perform more in-depth studies to clarify this point.

This investigation focuses on the application of SML algorithms to forecast the CS of GPC. Four types of SML approaches were used in this study: DT, GEP, BR, and RF. Their predictive ability was evaluated and compared using statistical checks and R^2^ values, and the results were published in JML. The selection of two individual ML techniques (DT and GEP) and two ensemble ML algorithms (BR and RF) was due to their popularity in giving a high precision towards the prediction of required outcomes. Additionally, two types of ML techniques (individual and ensemble) also show a better understanding to the readers for comparing with one another. The validity of each technique was also determined using k-fold cross-validation (KFCV) checks and error dispersions. DT and GEP are individual SML techniques, while BR and RF are ensembled SML algorithms [14,44]. In this study, the novel feature is that it investigates the CS of GPC by employing both single and ensemble SML strategies to forecast the CS of geopolymer concrete, whereas experimentally based studies necessitate a significant amount of human effort as well as expensive and time-consuming experimental procedures. Employing modern techniques such as SML will benefit the construction field by addressing the aforesaid issues. Because a variety of parameters, such as precursor materials, activator solution, aggregate quantity, and so on, influence the strength of GPC, it is difficult to examine their combined impact using experimental techniques. ML techniques are capable of identifying the cumulative influence of their components with minimal effort. ML methods require a data set, which may be gathered from previous research since several investigations have been conducted to determine the strength of GPC. The data collection can then be used to train ML models and anticipate material properties. Some recent studies have been performed to estimate the strength of GPC using ML methods with a limited number of input parameters and data points. For example, Dao et al. [47] predicted the CS of GPC using ML techniques with 3 input variables and 210 data points. Similarly, another study used 4 input variables and 210 data points [48]. The present research employed 9 input variables and 371 data points to forecast the CS of GPCs using different ML techniques to compare their performance. In addition, the outcomes of this study are compared with past relevant studies. It is expected that using a higher number of input variables and data points will result in the superior precision of ML techniques. The goal of this study is to determine the most appropriate ML approach for estimating the CS of GPC using forecasted outcomes and the influence of various factors on GPC strength.

## 2. Research Methods

### 2.1. Data Retrieval and Analysis

**Figure 3:**
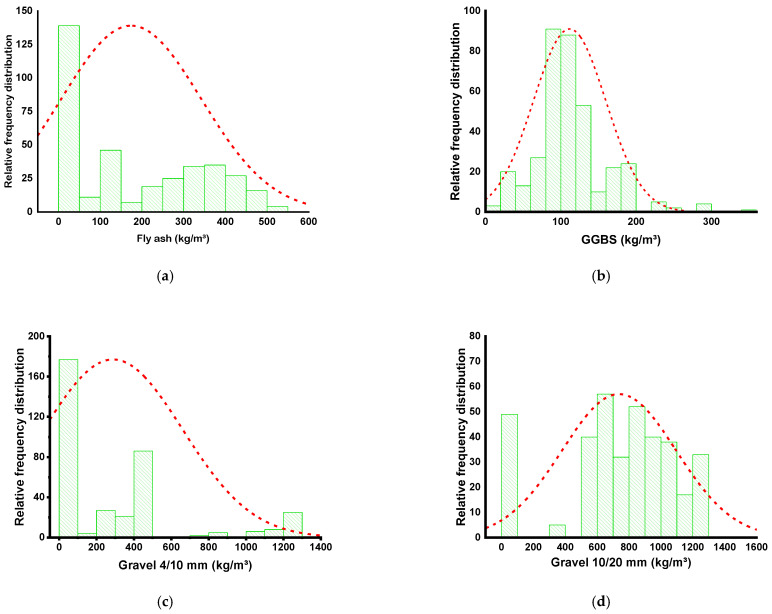

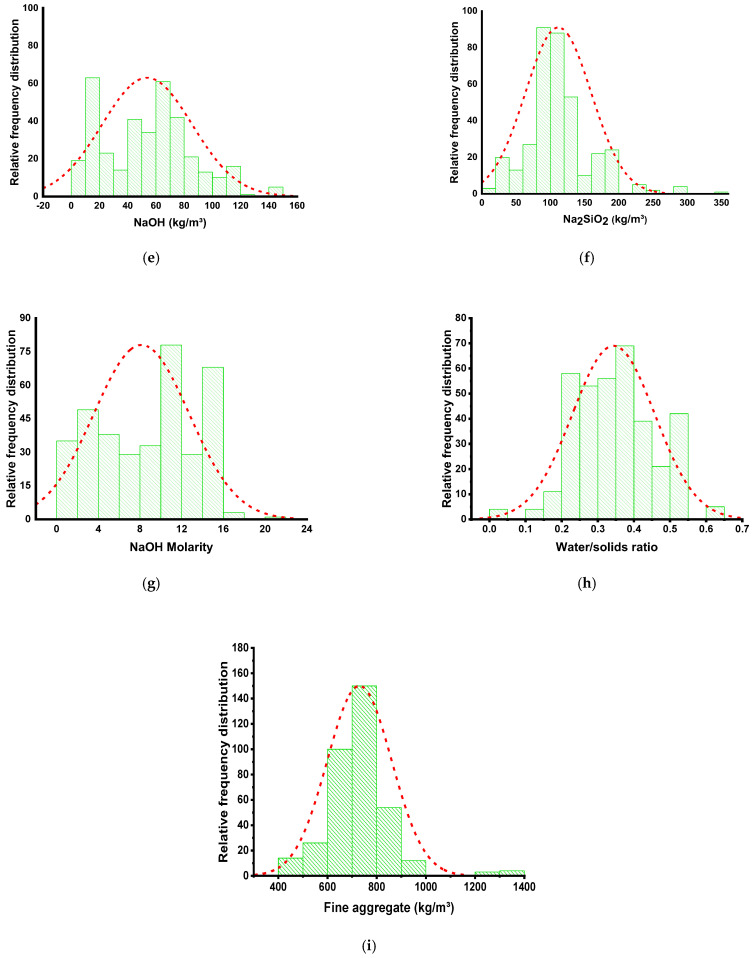
Comparable dispersal of the frequency for the variables: (**a**) fly ash; (**b**) GGBS; (**c**) 4/10 size gravel mm; (**d**) 10/20 size gravel mm; (**e**) NaOH; (**f**) Na_2_SiO_3_; (**g**) NaOH molarity; (**h**) water/solids ratio; and (**i**) fine aggregate.

SML algorithms need a diverse set of input variables to generate the desired output [49]. The CS of GPC was projected by retrieving data from the published articles (see Table S1 in Supplementary Materials). The number of articles published on the usage of similar materials for the CS of GPC was analyzed. While most articles analyzed various properties of GPC, this research required that just CS-based data points be obtained in a sufficient quantity to run the models. The models took nine variables as inputs, including fly ash, GGBS, Na_2_SiO_3_, NaOH, fine aggregate, two types of gravels gravel, i.e., 4/10 mm, and 10/20 mm, NaOH molarity, and water/solids ratio. They took CS as a single output variable for each selected model. The number of input variables and data sets significantly affects the output of the model [50]. A total of 371 data points was incorporated for running SML techniques in the current study. Table 1 presents a descriptive statistical assessment of all input factors. Figure 3 illustrates the relative occurrence dispersion of all variables utilized in the analysis. It illustrates the number of overall observations that correspond to each value or set of values. It is inextricably linked to a probability distribution, a statistical concept that is commonly utilized.

### 2.2. Analysis of Techniques Employed

**Figure 4:**
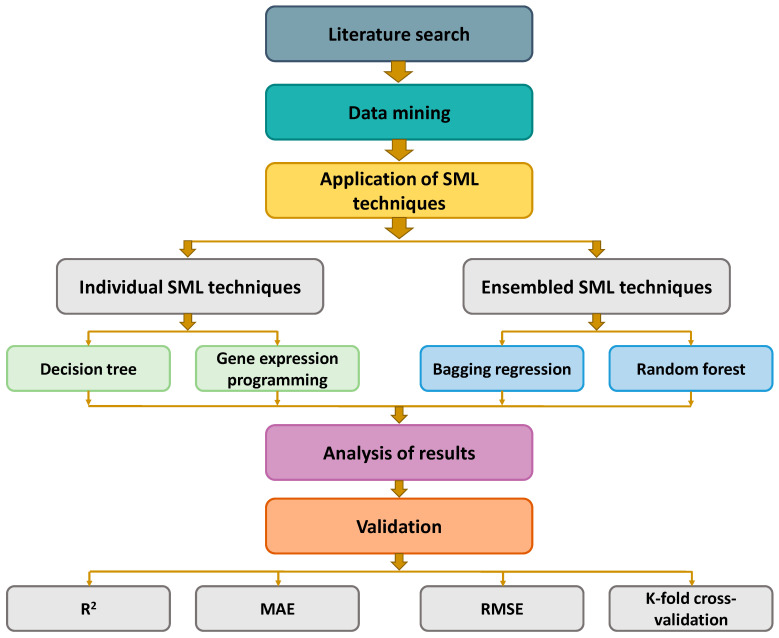
Flowchart of research methodology.

Individual SML techniques (DT and GEP) and ensemble SML techniques (BR and RF) were employed to achieve the objectives of this study, employing Python coding via the software named Anaconda Navigator and GEP. To execute the DT, BR, and RF models, Spyder (Version 4.3.5) was picked from the software. Typically, these types of algorithms are employed to forecast needed results depending on input parameters. These methods can predict the temperature impact, strength, and durability characteristics of materials [51,52]. The number of input variables was nine, and the output variable was one (i.e., CS) employed throughout the modeling phase. All models’ R^2^ reading for the anticipated result indicated their validity/precision. The R^2^, also called the coefficient of determination, estimates the extent of variation in a response variable that is given by a model. In other terms, it expresses the model’s fitness to the data quantitatively. A number close to 0 implies that fitting the mean is equivalent to fitting the model, whereas a result near 1 indicates that the model and data are virtually perfectly fitted [14]. The SML techniques employed in this study are described in the below sub-sections. Furthermore, to validate models, statistical, KFCV, and error investigation were employed on all techniques, i.e., RMSE and MAE. Additionally, sensitivity analysis was carried out to assess the contribution of each input variable toward the estimation of results. Figure 4 illustrates the research methodology in the form of a flowchart.

#### 2.2.1. Decision Tree ML Technique

**Figure 5:**
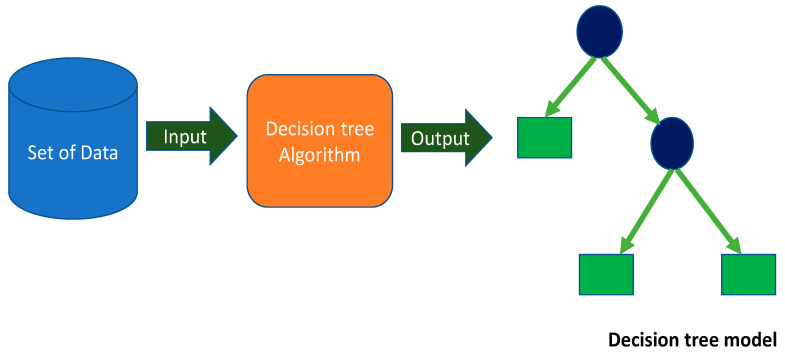
Decision tree schematic representation.

The DT method is a family member of the supervised learning algorithm. In addition to solving regression-type problems, it can also solve classification problems. The aim is to build a model that accurately forecasts the value of a target variable by inferring basic decision rules from the dataset. The purpose also includes generating a training model capable of anticipating the class or value of a target variable by inferring basic decision rules from prior data (training data). In decision trees, the process starts by forecasting a class label for a record at the tree’s root. The result of the root attribute is compared to those of the record’s attribute. It adopts the branch corresponding to that value and moves to the next node based on the relevant comparison. The detailed process of modeling with DT can be seen in Figure 5.

#### 2.2.2. Gene Expression Programming Technique

**Figure 6:**
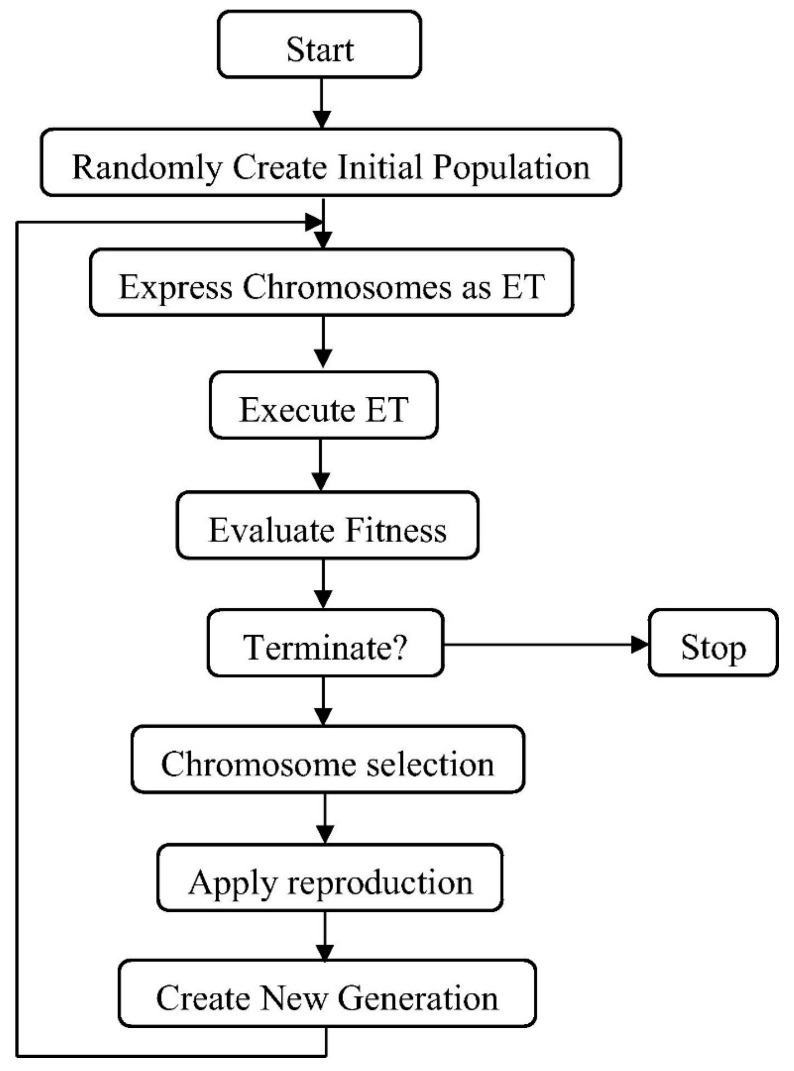
Schematic representation of gene expression programming [54].

Ferreira [53] invented GEP as a subfield of genetic programming. It is composed of five distinct elements, including fitness function, terminal set, function set, terminal condition, and control parameters. The GEP approach uses a fixed-length role string to acquire the solution, whereas the genetic programming technique utilizes a parse tree structure that can change in length during the course of computer programming. The GEP makes it exceedingly simple to create genetic variation because of the chromosomal level’s genetic mechanism. Additionally, because GEP is multi-genic, it enables the building of complicated and nonlinear programs comprising several subprograms. Figure 6 illustrates the GEP algorithm schematically. The process begins by randomly generating a chromosome of stable length for each growing program. Consequently, the chromosomes are confirmed, and each individual’s fitness is appraised. After that, individuals are picked for reproduction on the basis of their fitness findings. The process is repeated with each new individual until a solution is discovered. This method provides a transition in the population by executing genetic processes on the specified program, such as rotation, mutation, and crossover [54]. GEP is a transforming process for the creation of computer modules and programs. As with chromosomes, these programs often have a tree formation that can change dimensions (size/shape). Therefore, GEP can be significantly more effective than adaptive techniques as a genotype–phenotypic system. Karva is GEP’s programming language, and it is similar to the LISP languages. GEP provides a number of advantages over other standard regression techniques, which generate functions first and then evaluate them. However, GEP makes no provision for predefined functions.

#### 2.2.3. Bagging Regressor Approach

**Figure 7:**
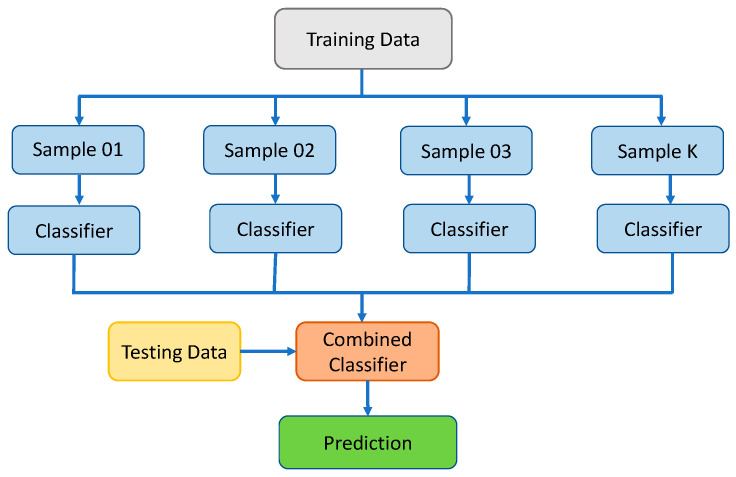
Schematic representation of bagging regressor technique.

BR is a comparable SML technique that compensates for the prediction model’s variance during the training stage by improving it with Supplementary Materials. This result is established on an asymmetric selection strategy that makes use of data exchange from the original set. Utilizing sampling with substitute, some observations may be reiterated in each new testing dataset, allowing for greater accuracy. During the BR process, each constituent has an equal probability of being included in the new dataset, regardless of its importance. There is no influence on the forecasting force of a training set that is larger in size than the training set. It is also possible to considerably reduce the variation by fine-tuning the estimate to obtain the desired conclusion. For subsequent model training, each of these data sets is commonly utilized to supplement the others. Using an ensemble of numerous models, the mean of all predictions from each model is used to create this ensemble. In regression, the prediction might be the average or mean of the estimates from a number of different models [55]. Twenty sub-models are employed to optimize the DT using BR to obtain an adamant output result. Figure 7 depicts the BR algorithm’s flow chart, which details the procedure until the desired output is obtained.

#### 2.2.4. Random Forest (RF) Technique

**Figure 8:**
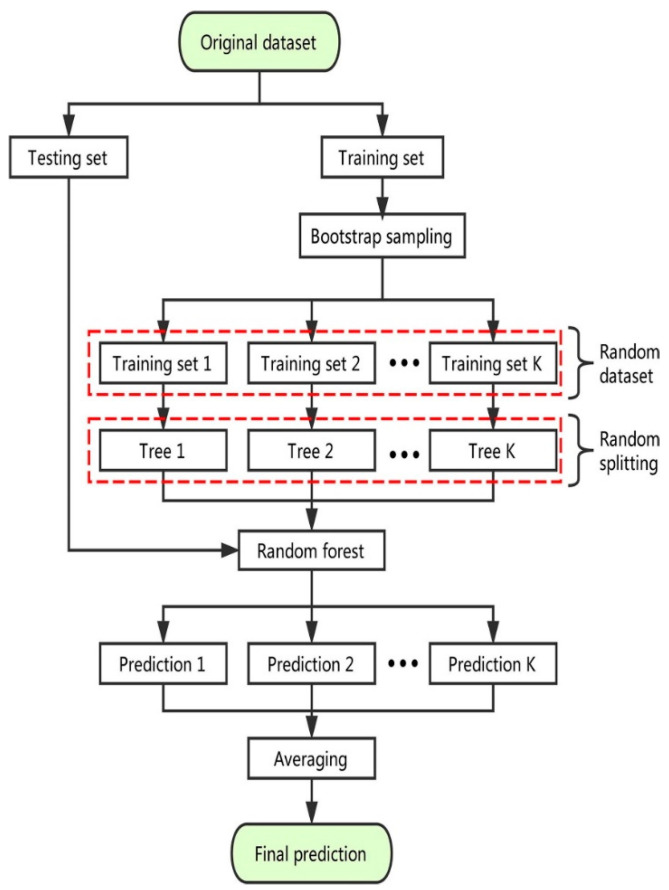
The step-by-step execution process of the random forest algorithm [56].

RF is a type of strategy for supervised learning. It builds a “forest” out of an ensemble of DTs, which are normally trained with the “bagging” method. The bagging technique’s basic assumption is that merging several learning models enhances the result. RF combines many DTs to generate a more precious and consistent prediction. One important advantage of RF is that it can also be utilized to solve regression- and classification-type problems, which constitute most ML tasks these days. The hyperparameters of an RF are very comparable to those of a DT or a bagging classifier. There is no need to combine a DT with a bagging classifier as the classifier class of RF may be employed directly. It may also perform regression problems using RF by utilizing the algorithm’s regressor. The process of RF is also explained via graphical representation, as shown in Figure 8.

## 3. Analysis of Results

### 3.1. Model Result of the Decision Tree

**Figure 9:**
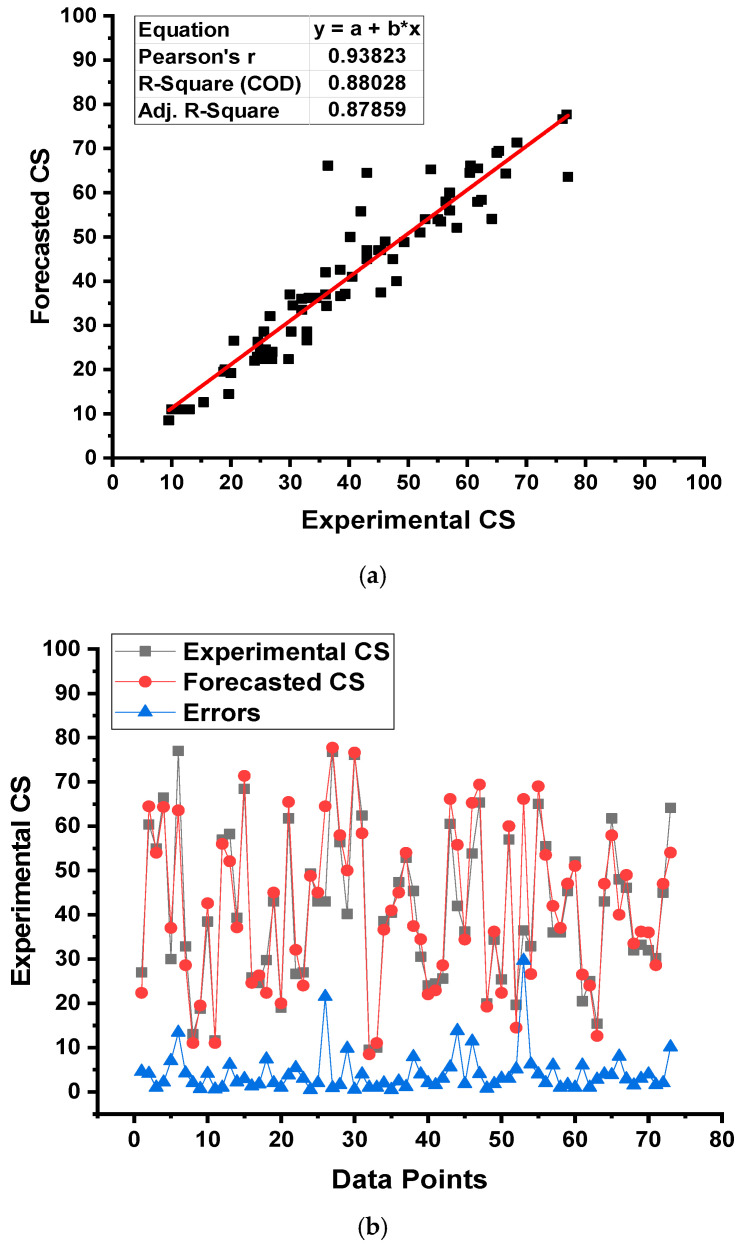
Decision tree model: (**a**) relationship among experimental and forecasted outcomes and (**b**) distribution of experimental and forecasted outcomes.

The outcomes of the DT model for the CS of GPC have been displayed in Figure 9. The relationship between the experimental and forecasted results is shown in Figure 9a. The DT approach produced output with an acceptable degree of precision and a small difference amongst the experimental and forecasted results. The R^2^ of 0.88 confirms that the DT model has a reasonable degree of accuracy in anticipating the CS of selected concrete. Figure 9b demonstrates the dispersion of the experimental, anticipated, and result of the differences between the real and predictions for the DT model. By analyzing these values, it was reported that lower, average, and maximum results (values) were 0.50, 4.14, and 29.68 MPa. In addition, the percentage distribution of the said results was evaluated, and it was reported that 9.6% were less than 1 MPa, 43.8% between 1 and 3 MPa, 21.9% between 3 and 5 MPa, and 16.4% between 5 and 10 MPa, while only 8.2% data was found larger than 10 MPa. This dispersion of these values also indicates a satisfactory performance of the DT model.

### 3.2. Gene Expression Programming Model

**Figure 10:**
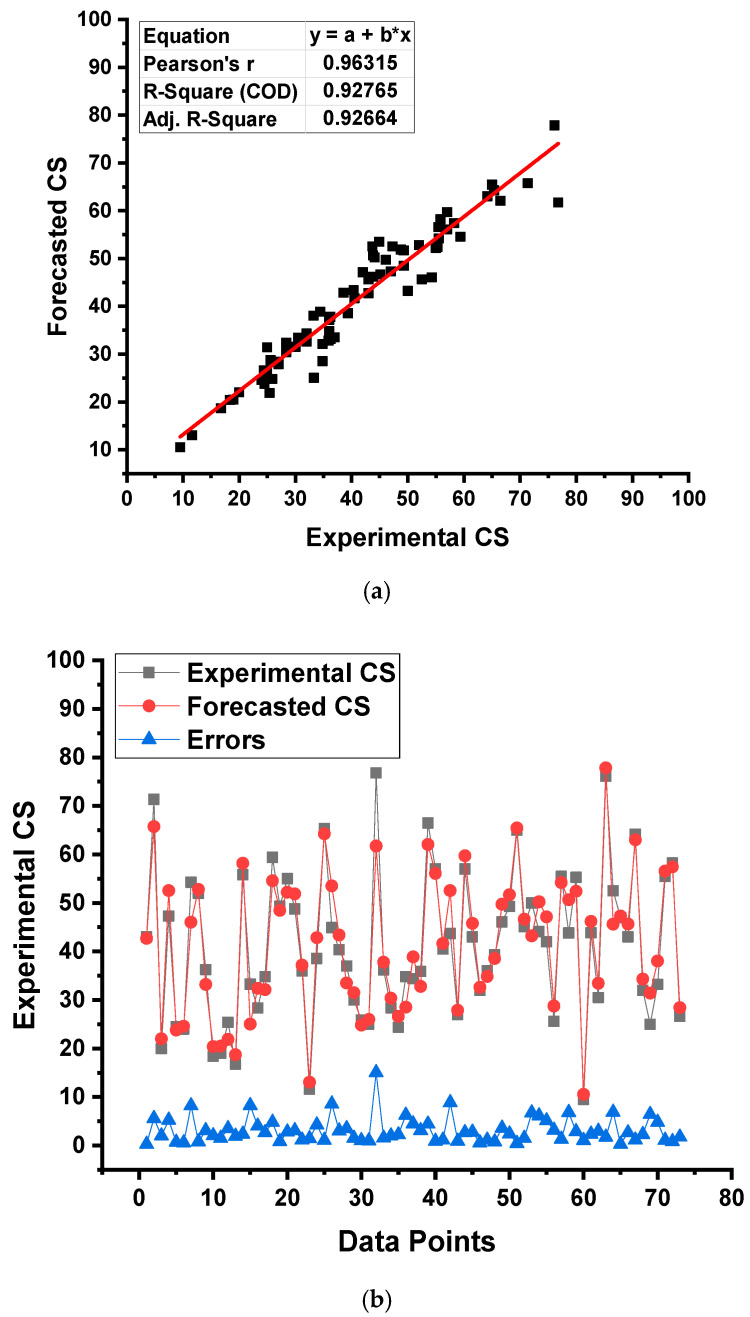
Gene expression programming model: (**a**) relationship among experimental and forecasted outcomes and (**b**) distribution of experimental and forecasted outcomes.

Figure 10a,b compare the experimental and forecasted outputs of the GEP model. Figure 10a shows the relationship amongst the result of the CS obtained from the experimental approach and the result derived from the selected model, with an R^2^ value of 0.93, implying the GEP model’s higher precision than the DT model in estimating the CS of the selected concrete. The distribution of the experimental, anticipated, and different values of real data and predictions for the GEP model are depicted in Figure 10b. It was noticed that the lowest, average, and maximum error values were 0.28, 3.10, and 15.07 MPa, respectively. This difference (errors) between the actual and predicted values was reported as 16.44% less than 1 MPa, 45.21% between 1 and 3 MPa, and 19.18% between 3 and 5 MPa, while the limited percentage (19.18%) was more than 5 MPa. This limited result of the errors also manifests the GEP model’s superior exactness to the DT model.

### 3.3. Bagging Regressor Model

**Figure 11:**
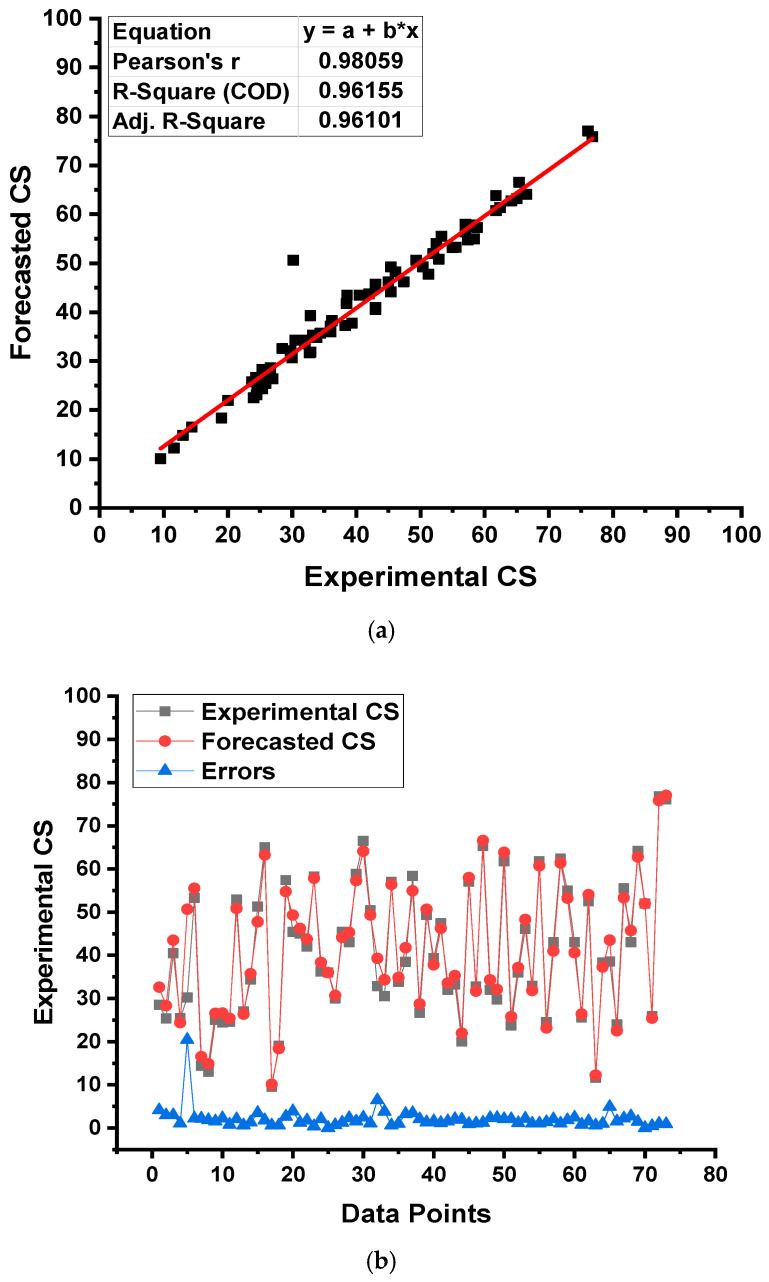
Bagging regressor model: (**a**) relationship among experimental and forecasted outcomes and (**b**) distribution of experimental and forecasted outcomes.

Figure 11 describes the results obtained from the BR ML model for the CS estimation of the concrete. Figure 11a gives a reflection of the link among the results obtained from the experiments in the laboratory and the outcomes generated from the selected ML model. The BR algorithm produced the output with the highest degree of precision and a minimal divergence amongst the experimental and projected results. The R^2^ of 0.96 indicates that the BR model is highly precise at forecasting the CS of the selected concrete. The spreading of the experimental, anticipated, and the difference between them for the BR model is illustrated in Figure 11b. The minimum, average, and highest error values for the data set were noted to be 0.03, 2.04, and 20.45 MPa, respectively. This distribution of the error values was noted as 21.9% less than 1 MPa and 65.8% between 1 and 3 MPa, while the limited percentage (12.3%) exceeded 3 MPa. The distribution of error values also implies the best precision of the BR model in predicting outcomes.

### 3.4. Random Forest Model

**Figure 12:**
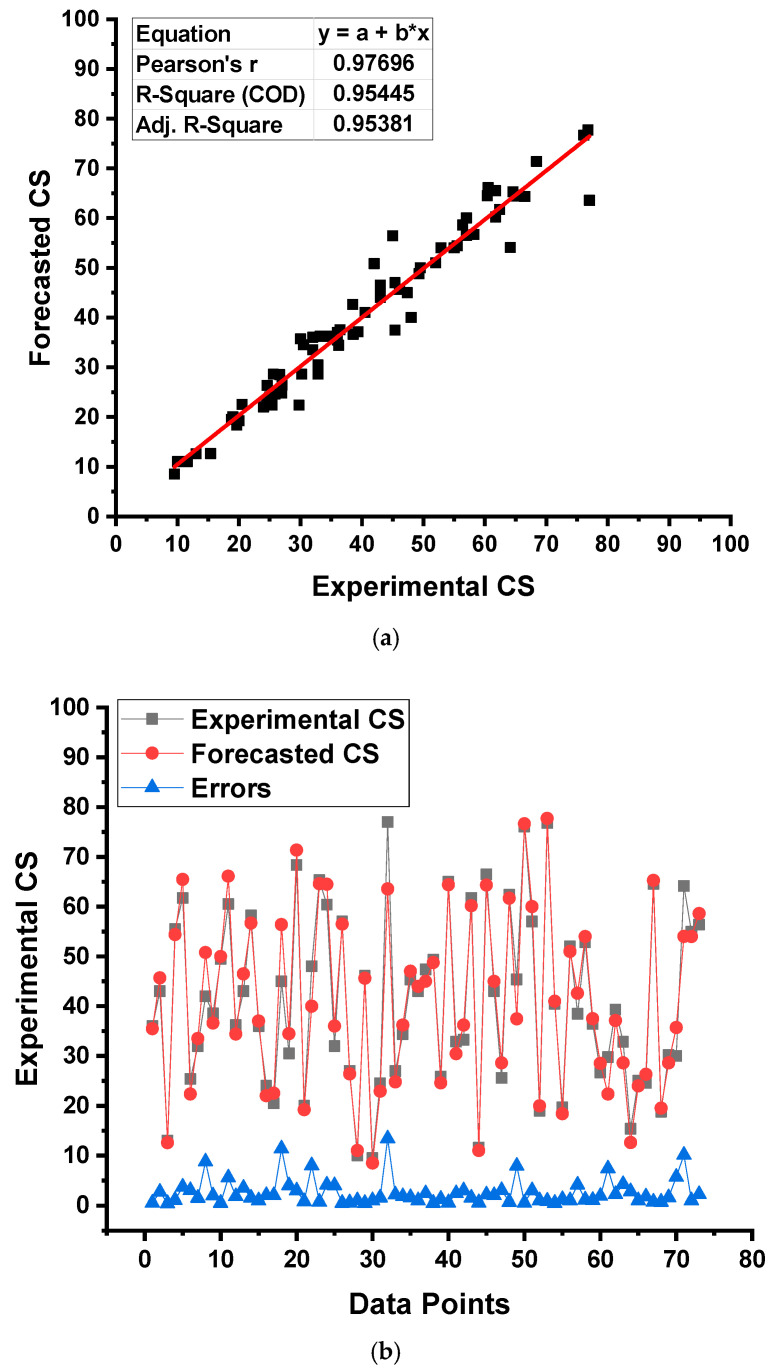
Result derived from the random forest model: (**a**) Relationship between results from the experimental approach and results from the model; (**b**) Distribution of experimental and forecasted outcomes.

A comparable depiction of the outcomes of the RF model has been presented in Figure 12. An R^2^ value of 0.95 in Figure 12a shows a comparable performance of the RF model to the BR model. The dispersion of experimental CS results, forecasted results, and differences of those for the RF model are shown in Figure 12b. The minimum, average, and higher error values were found to be 0.40, 2.59, and 13.42 MPa, respectively. Error-values dispersal was found as 23.29% less than 1 MPa and 50.68% between 1 and 3 MPa, and only 26.03% surpassed 3 MPa. This distribution shows the RF model’s better predictive accuracy. Hence, it can be concluded that the ensembled SML techniques had greater accuracy than the individual SML techniques.

## 4. Validation

**Figure 13:**
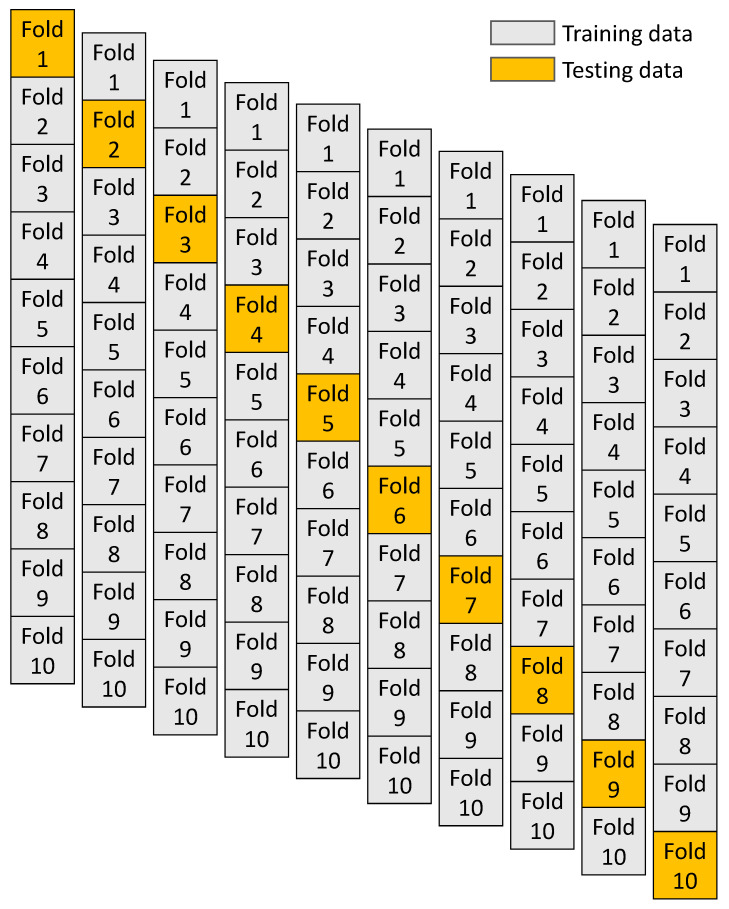
K-fold cross-validation procedure.

The employed models were validated using statistical checks and the KFCV technique. Mostly, the KFCV procedure is used to generate the validity of the model [44], during which related data is randomly disseminated and separated into 10 groups. Nine from all will be employed for training the selected model, and the remaining single one will be allocated to validate the said model, as shown in Figure 13. Eighty percent of the database was selected for training these models, while twenty percent was used to evaluate these models. The model is normally considered more precious in terms of predictions when the result of the errors (MAE and RMSE) becomes minimal and shows the R^2^ high. Additionally, the procedure must be performed ten times until a suitable outcome is obtained. This thorough process contributes toward the higher precision level of the model for forecasting the required outcomes. Moreover, as listed in Table 2, all employed ML models were subjected to statistical evaluation through errors such as MSE and RMSE. These checks also validated the increased accuracy of the BR model because of its reduced error of readings as opposed to the other techniques. The models’ predictive performance was evaluated through statistical evaluation in line with Equations (1) and (2), obtained from the past studies [57,58].

(1) $$\mathrm{MAE}=\frac{1}{n}\overset{n}{\underset{i=1}{\sum }}\left|x_{i}-x\right|$$

(2) $$\mathrm{RMSE}=\sqrt{\sum \frac{{\left(y_{pred}-y_{ref}\right)}^{2}}{n}}$$

where n = total dataset, x $y_{ref}$ = reference values in the dataset, and $x_{i}$, $y_{pred}$ = projected values from ML methods.

**Figure 14:**
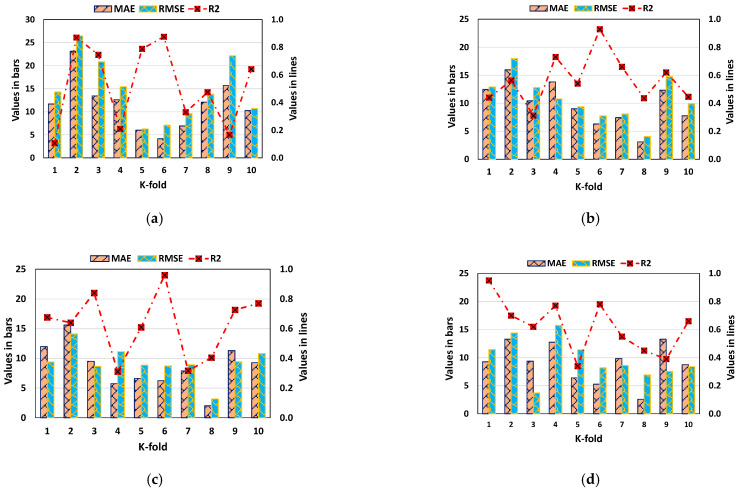
K-fold cross-validation statistical representation: (**a**) decision tree; (**b**) gene expression programming; (**c**) bagging regressor; and (**d**) random forest.

In order to assess the KFCV, R^2^, MAE, and RMSE calculated, and their results for the DT, GEP, BR, and RF models are depicted in Figure 14. As shown in Figure 14a, the minimum, average, and maximum MAE values for the DT model were 4.14, 11.59, and 23.11, respectively. By comparison, the minimum, average, and maximum MAE values for the GEP model were 3.10, 9.86, and 15.95, respectively (Figure 14b), whereas the minimum, average, and maximum MAE values for the BR model were 2.04, 8.63, and 15.61, respectively (Figure 14c). Moreover, the lowest, average, and highest MAE values for the RF model were 2.59, 9.08, and 13.28, respectively (Figure 14d). When the RMSE values were compared, the DT, GEP, BR, and RF model’s average RMSE values were 14.66 and 10.83, 9.32, and 9.63, respectively. However, the average R^2^ results for DT, GEP, BR, and RF were found to be 0.52, 0.57, 0.63, and 0.62, respectively. The BR model with the lowermost error values and a superior R^2^ value is the most accurate model compared to the others in predicting the compressive strength of the said concrete. Table 3 contains the results of the k-fold analysis for each of the models used, along with their MAE, RMSE, and R^2^ values.

## 5. Sensitivity Analysis

**Figure 15:**
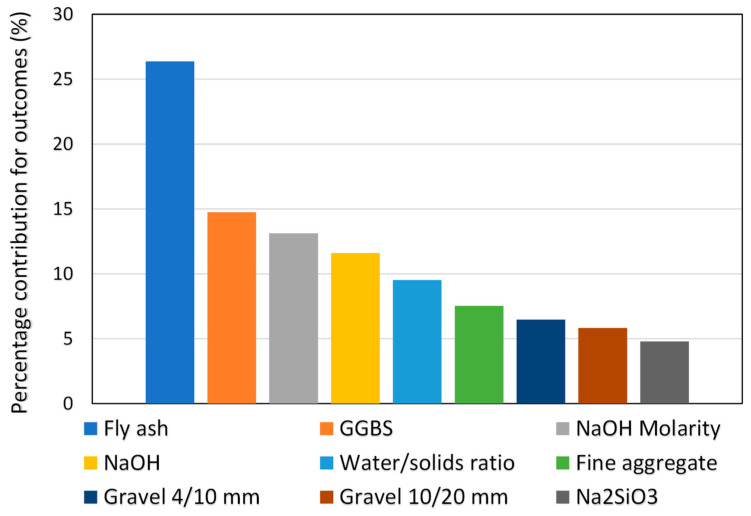
Input variables contribution to predicting outcomes.

This assessment intends to ascertain the impact of input variables on the CS forecasting of GPC. The input variables significantly affect the expected outcome [59]. The impact of each input on the CS estimate of GPC is seen in Figure 15. The investigation found that fly ash was the most important element with a contribution of 26.4%, followed by GGBS with a contribution of 14.7% and NaOH molarity with a contribution of 13.1%. The other parameters contributed the least to the prediction of concrete’s strength, with NaOH having 11.6%, water/solids ratio 9.5%, fine aggregate 7.5%, gravel 4/10 mm 6.5%, gravel 10/20 mm 5.8%, and Na_2_SiO_3_ 4.8% contribution. Equations (2) and (3) were used to determine the effect of each input variable on the model’s output.

(3) $$N_{i}=f_{max}\left(x_{i}\right)-f_{min}\left(x_{i}\right),$$

(4) $$S_{i}=\frac{N_{i}}{\sum_{j\;-\;i}^{n}N_{j}},$$

where $f_{max}\left(x_{i}\right)$ and $f_{min}\left(x_{i}\right)$ are the higher and lower of the result over the $i^{th}$ outcome, respectively, and others are constant. The $S_{i}$ is the contribution proportion (achieved) for the selected variable.

## 6. Discussions

This study was performed to expand the knowledge on the application of modern techniques to estimate the strength of GPC. This study will benefit the construction sector with rapid and cost-effective techniques for the prediction of material properties. In addition, the selection and application of this type of concrete (GPC) in construction will be achieved earlier using these techniques to promote eco-friendly construction. This research validates how SML techniques can be effectively employed to forecast the strength of the said concrete. Four different types of SML approaches were introduced in the study such as DT, GEP, BR, and RF. The GEP and DT belong to the individual ML algorithms, while BR and RF are the types of ensemble ML approaches. The precision level of each technique towards the anticipating was evaluated to determine which approach is the most effective predictor. The BR model gave a more precise result, with an R^2^ of 0.96, than the RF, GEP, and DT models, which yielded R^2^ values of 0.95, 0.93, and 0.88, respectively. The high precision of the ensemble ML approaches is due to the execution process of these models. These models normally split themselves into the 20 sub-models. The model with a high R^2^ value was then selected and incorporated for further findings. In addition, statistical analysis and the KFCV approach were used to validate the performance of all models. The more reduced the error levels, the more precise the model. However, measuring and suggesting the ideal SML model for predicting the required outputs through a number of subjects is challenging, as each model’s success is largely dependent on the input parameters and data points employed to execute the algorithm. Ensembled SML approaches, on the other hand, frequently take advantage of the weak learner by constructing sub-models that can be trained on data and adjusting to optimize the R^2^ value.

**Figure 16:**
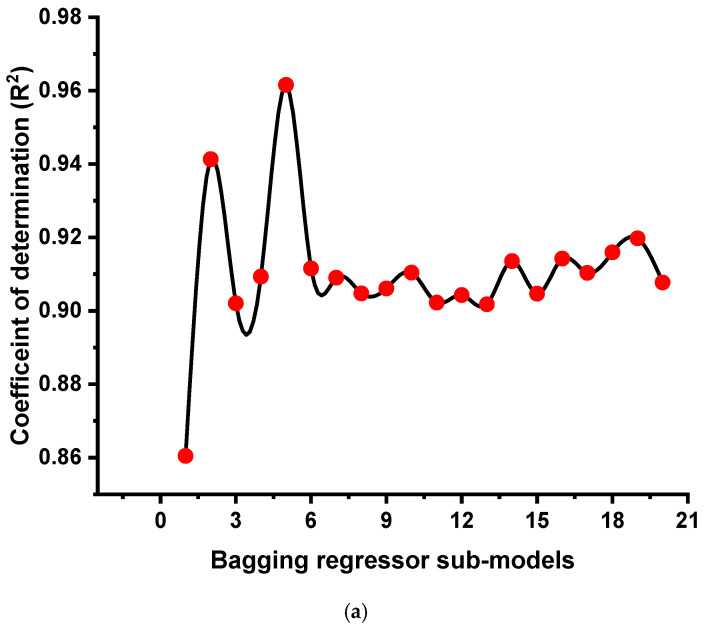

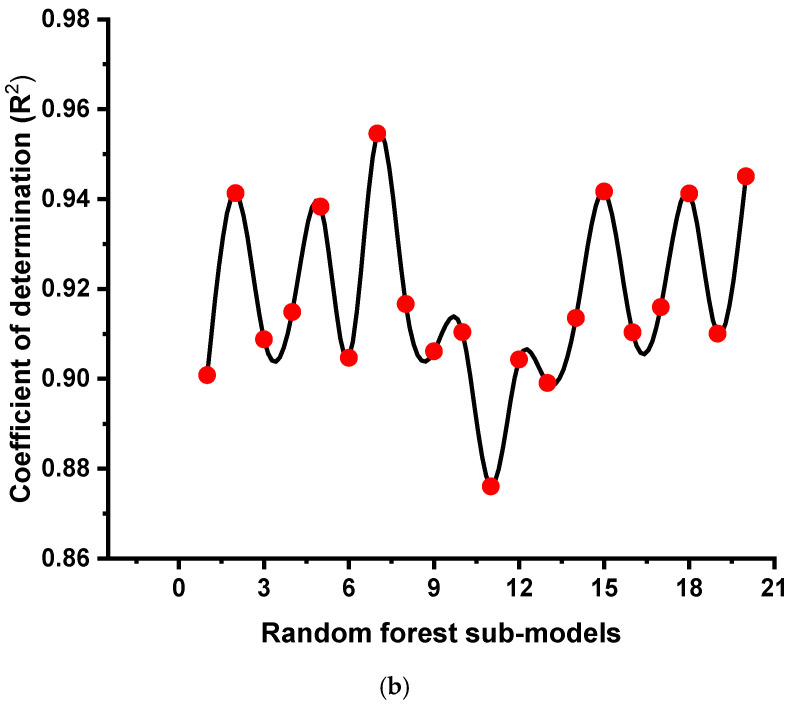
R^2^ result of the sub-models: (**a**) bagging regressor and (**b**) random forest.

Figure 16 depicts the dispersion of R^2^ values for BR and RF sub-models. For BR sub-models, the lower, average, and maximum R^2^ values were 0.860, 0.911, and 0.962, respectively (Figure 16a), whereas for RF sub-models, the minimum, average, and maximum R^2^ were 0.876, 0.918, and 0.955, respectively (Figure 16b). These numbers give the reflection that the BR and RF sub-models have almost similar values and a good level of exactness in estimating the required results. According to the recent literature, as reported by Ahmad et al. [59], the maximum precision towards the prediction of concrete strength from the various employed ML approaches was shown by the BR model. The employed techniques such as DT and ANN’s R^2^ values were reported as 0.83 and 0.82, respectively, and the precision level of the models presented in this study was noted to be higher, giving R^2^ values equal to 0.95 and 0.96, respectively. Additionally, Song et al. [60] also compared the accuracy level of different ML algorithms for predicting the strength of concrete. The GEP (0.86), ANN (0.81), and DT (0.75) accuracy levels for predicting the required outcome were also less than the ML approaches used in this study. Dao et al. [47] used particle swarm optimization (PSO)-based, adaptive network-based, fuzzy inference system (PSOANFIS) and a genetic algorithm (GA)-based, adaptive network-based, fuzzy inference system (GAANFIS) for the prediction of geopolymer concrete. It was also reported that the algorithms used in this study show better performance than PSOANFIS, which gives an R^2^ value equal to 0.93, and GAANFIS gives this value equal to 0.92. Similarly, it was also reported [48] that the adaptive neuro-fuzzy inference (ANFIS) and artificial neural network (ANN) shows better performance in term of forecasting the strength of concrete but lesser than the employed ML techniques in the study. Moreover, a sensitivity analysis was carried out to discover the influence of each input variable on GPC’s anticipated CS. It is also noted that the model’s accuracy can be influenced by the variables used in the dataset and the size of the dataset. The sensitivity analysis reported the contribution of each of the nine inputs to the predicted output. The top three contributing input variables were determined to be fly ash, GGBS, and NaOH molarity.

## 7. Conclusions

The intent of the research was to apply individual and ensembled supervised machine learning (SML) methods to investigate the compressive strength (CS) of geopolymer concrete (GPC). To predict outcomes, two individual techniques, decision tree (DT) and gene expression programming (GEP), and two ensembled techniques, bagging regressor (BR) and random forest (RF), were used. This research has yielded the following conclusions:

- Ensembled SML approaches were more accurate than individual SML techniques at predicting the CS of GPC, with the BR model having the best accuracy. The coefficients of determination (R^2^) were 0.96, 0.95, 0.93, and 0.88 for BR, RF, GEP, and DT models, respectively. All models produced findings within a satisfactory range and had little deviation from the real data.
- Checks from the statistics and K-fold cross-validation confirmed the model’s performance as well. In addition, these checks proved that the BR model outperformed the other models evaluated.
- Sensitivity analysis revealed that fly ash, GGBS, NaOH molarity, water–to–solids ratio, sand, gravel with 10/20 mm in size, NaOH, gravel with 4/10 mm in size, and Na_2_SiO_3_ contributed 26.4%, 14.7%, 13.1%, 11.6%, 9.5%, 7.5%, 6.5%, 5.8%, and 4.8%, respectively, for the anticipation of output.
- This study will benefit the construction industry by producing quick and cost-effective approaches for predicting the strengths of materials. Additionally, utilizing these ways to promote more eco-friendly construction will expedite the adoption of GPC in construction projects.

It is further recommended that the number of data points can be increased with the help of experimental approaches in the laboratory. Moreover, the experimental tests can also be performed on the material used, such as geopolymer, concrete, aggregate, and admixtures, to investigate their effects during the execution process of employed models.

## Figures and Tables

**Table 1:** Explanatory statistics of the parameters employed to run the selected models.

Parameter	Fly Ash (kg/m^3^)	GGBS (kg/m^3^)	Na_2_SiO_3_ (kg/m^3^)	NaOH (kg/m^3^)	Fine Aggregate (kg/m^3^)	Gravel 4/10 mm (kg/m^3^)	Gravel 10/20 mm (kg/m^3^)	Water/Solids Ratio	NaOH Molarity
Mean	174.34	225.15	111.66	53.74	729.88	288.39	737.37	0.34	8.14
Mode	0	0	108	64	651	0	0	0.53	10
Median	120	300	108	56	728	208	789	0.34	9.2
Standard Deviation	167.95	162.27	48.16	31.91	130.97	372.31	358.55	0.11	4.56
Sum	63,286.04	81,728.05	40,532.68	19,508.75	264,947.79	104,684.28	267,664.93	124.78	2955.11
Range	523	450	324	143.5	901	1293.4	1298	0.63	19
Maximum	523	450	342	147	1360	1293.4	1298	0.63	20
Minimum	0	0	18	3.5	459	0	0	0	1
Standard Error	8.82	8.52	2.53	1.67	6.87	19.54	18.82	0.01	0.24

**Table 2:** Statistical outcomes for the employed models.

SML Technique	MAE	RMSE
Decision tree	4.136	6.256
Gene expression programming	3.102	4.049
Bagging regressor	2.044	3.180
Random forest	2.585	3.702

**Table 3:** Outcomes of KFCV for all the models employed.

K-Fold	DT			GEP			BR			RF		
	MAE	RMSE	R^2^	MAE	RMSE	R^2^	MAE	RMSE	R^2^	MAE	RMSE	R^2^
1	11.67	14.28	0.11	12.48	12.90	0.44	11.99	9.40	0.68	9.26	11.43	0.95
2	23.11	26.44	0.87	15.95	17.98	0.56	15.61	14.10	0.64	13.28	14.38	0.70
3	13.44	20.82	0.75	10.44	12.78	0.31	9.52	8.65	0.84	9.37	3.70	0.62
4	12.61	15.43	0.21	13.79	10.77	0.73	5.75	11.14	0.31	12.75	15.74	0.77
5	6.00	6.26	0.79	8.99	9.37	0.54	6.63	8.85	0.61	6.40	11.38	0.34
6	4.14	7.10	0.88	6.29	7.73	0.93	6.25	8.74	0.96	5.27	8.19	0.78
7	6.92	9.62	0.33	7.45	8.08	0.66	7.90	8.93	0.32	9.84	8.60	0.55
8	12.10	13.81	0.47	3.10	4.05	0.44	2.04	3.18	0.41	2.59	6.94	0.45
9	15.68	22.15	0.17	12.32	14.74	0.62	11.33	9.45	0.73	13.28	7.55	0.39
10	10.25	10.69	0.64	7.75	9.90	0.45	9.28	10.81	0.77	8.75	8.38	0.66

## Data Availability

The data used in this research has been properly cited and reported in the main text.

## Supplementary Materials

The following supporting information can be downloaded at: https://www.mdpi.com/article/10.3390/gels8050271/s1, Table S1: Data used for modeling.

## Abbreviations

BR	Bagging regressor
CS	Compressive strength
DT	Decision tree
OPC	ordinary Portland cement
GPC	geopolymer concrete
GEP	Gene expression programming
KFCV	K-fold cross-validation
MAE	mean absolute error
MSE	Mean square error
ML	Machine learning
RF	Random forest
RMSE	Root mean square error
SML	Supervised machine learning

## References

[1] Chu S.H., Ye H., Huang L., Li L.G. (2021). Carbon fiber reinforced geopolymer (FRG) mix design based on liquid film thickness. Constr. Build. Mater..

[2] Homayoonmehr R., Ramezanianpour A.A., Mirdarsoltany M. (2021). Influence of metakaolin on fresh properties, mechanical properties and corrosion resistance of concrete and its sustainability issues: A review. J. Build. Eng..

[3] Ahmad M.R., Chen B., Haque M.A., Oderji S.Y. (2020). Multiproperty characterization of cleaner and energy-efficient vegetal concrete based on one-part geopolymer binder. J. Clean. Prod..

[4] Khan M., Ali M. (2016). Use of glass and nylon fibers in concrete for controlling early age micro cracking in bridge decks. Constr. Build. Mater..

[5] Khan M., Cao M., Ali M. (2020). Cracking behaviour and constitutive modelling of hybrid fibre reinforced concrete. J. Build. Eng..

[6] Khan M., Ali M. (2018). Effect of super plasticizer on the properties of medium strength concrete prepared with coconut fiber. Constr. Build. Mater..

[7] Khan M., Cao M., Chaopeng X., Ali M. (2021). Experimental and analytical study of hybrid fiber reinforced concrete prepared with basalt fiber under high temperature. Fire Mater..

[8] Teja K.V., Sai P.P., Meena T. Investigation on the behaviour of ternary blended concrete with scba and sf. Proceedings of the IOP Conference Series: Materials Science and Engineering.

[9] Gopalakrishnan R., Kaveri R. (2021). Using graphene oxide to improve the mechanical and electrical properties of fiber-reinforced high-volume sugarcane bagasse ash cement mortar. Eur. Phys. J. Plus.

[10] Yang H., Liu L., Yang W., Liu H., Ahmad W., Ahmad A., Aslam F., Joyklad P. (2022). A comprehensive overview of geopolymer composites: A bibliometric analysis and literature review. Case Stud. Constr. Mater..

[11] Bayasi Z., Zhou J. (1993). Properties of silica fume concrete and mortar. Mater. J..

[12] Li X., Qin D., Hu Y., Ahmad W., Ahmad A., Aslam F., Joyklad P. (2021). A systematic review of waste materials in cement-based composites for construction applications. J. Build. Eng..

[13] Cleetus A., Shibu R., Sreehari P.M., Paul V.K., Jacob B. (2018). Analysis and study of the effect of GGBFS on concrete structures. Int. Res. J. Eng. Technol..

[14] Ahmad A., Ahmad W., Aslam F., Joyklad P. (2022). Compressive strength prediction of fly ash-based geopolymer concrete via advanced machine learning techniques. Case Stud. Constr. Mater..

[15] Zhang P., Wang K., Li Q., Wang J., Ling Y. (2020). Fabrication and engineering properties of concretes based on geopolymers/alkali-activated binders-A review. J. Clean. Prod..

[16] Zakka W.P., Lim N.H.A.S., Khun M.C. (2021). A scientometric review of geopolymer concrete. J. Clean. Prod..

[17] Babu D.L.V. (2018). Assessing the performance of molarity and alkaline activator ratio on engineering properties of self-compacting alkaline activated concrete at ambient temperature. J. Build. Eng..

[18] Marvila M.T., Azevedo A.R.G.d., Vieira C.M.F. (2021). Reaction mechanisms of alkali-activated materials. Rev. IBRACON Estrut. Mater..

[19] Farooq F., Jin X., Javed M.F., Akbar A., Shah M.I., Aslam F., Alyousef R. (2021). Geopolymer concrete as sustainable material: A state of the art review. Constr. Build. Mater..

[20] Muttashar H.L., Ariffin M.A.M., Hussein M.N., Hussin M.W., Ishaq S.B. (2018). Self-compacting geopolymer concrete with spend garnet as sand replacement. J. Build. Eng..

[21] Farhan K.Z., Johari M.A.M., Demirboğa R. (2020). Assessment of important parameters involved in the synthesis of geopolymer composites: A review. Constr. Build. Mater..

[22] Hosan A., Haque S., Shaikh F. (2016). Compressive behaviour of sodium and potassium activators synthetized fly ash geopolymer at elevated temperatures: A comparative study. J. Build. Eng..

[23] Herath C., Gunasekara C., Law D.W., Setunge S. (2021). Long term mechanical performance of nano-engineered high volume fly ash concrete. J. Build. Eng..

[24] Ahmad W., Ahmad A., Ostrowski K.A., Aslam F., Joyklad P., Zajdel P. (2021). Application of Advanced Machine Learning Approaches to Predict the Compressive Strength of Concrete Containing Supplementary Cementitious Materials. Materials.

[25] Alyousef R., Ahmad W., Ahmad A., Aslam F., Joyklad P., Alabduljabbar H. (2021). Potential use of recycled plastic and rubber aggregate in cementitious materials for sustainable construction: A review. J. Clean. Prod..

[26] Khan M., Ali M. (2019). Improvement in concrete behavior with fly ash, silica-fume and coconut fibres. Constr. Build. Mater..

[27] Anjos M.A.S., Camões A., Campos P., Azeredo G.A., Ferreira R.L.S. (2020). Effect of high volume fly ash and metakaolin with and without hydrated lime on the properties of self-compacting concrete. J. Build. Eng..

[28] Mehta A., Ashish D.K. (2020). Silica fume and waste glass in cement concrete production: A review. J. Build. Eng..

[29] Tchakouté H.K., Rüscher C.H., Kong S., Ranjbar N. (2016). Synthesis of sodium waterglass from white rice husk ash as an activator to produce metakaolin-based geopolymer cements. J. Build. Eng..

[30] Ahmad A., Ahmad W., Chaiyasarn K., Ostrowski K.A., Aslam F., Zajdel P., Joyklad P. (2021). Prediction of Geopolymer Concrete Compressive Strength Using Novel Machine Learning Algorithms. Polymers.

[31] Reddy M.S., Dinakar P., Rao B.H. (2018). Mix design development of fly ash and ground granulated blast furnace slag based geopolymer concrete. J. Build. Eng..

[32] Van Deventer J.S.J., Provis J.L., Duxson P. (2012). Technical and commercial progress in the adoption of geopolymer cement. Miner. Eng..

[33] Jindal B.B. (2019). Investigations on the properties of geopolymer mortar and concrete with mineral admixtures: A review. Constr. Build. Mater..

[34] Wong C.L., Mo K.H., Alengaram U.J., Yap S.P. (2020). Mechanical strength and permeation properties of high calcium fly ash-based geopolymer containing recycled brick powder. J. Build. Eng..

[35] John S.K., Nadir Y., Girija K. (2021). Effect of source materials, additives on the mechanical properties and durability of fly ash and fly ash-slag geopolymer mortar: A review. Constr. Build. Mater..

[36] Pilehvar S., Cao V.D., Szczotok A.M., Carmona M., Valentini L., Lanzón M., Pamies R., Kjøniksen A.-L. (2018). Physical and mechanical properties of fly ash and slag geopolymer concrete containing different types of micro-encapsulated phase change materials. Constr. Build. Mater..

[37] Amlashi A.T., Abdollahi S.M., Goodarzi S., Ghanizadeh A.R. (2019). Soft computing based formulations for slump, compressive strength, and elastic modulus of bentonite plastic concrete. J. Clean. Prod..

[38] Sun J., Ma Y., Li J., Zhang J., Ren Z., Wang X. (2021). Machine learning-aided design and prediction of cementitious composites containing graphite and slag powder. J. Build. Eng..

[39] Milad A., Hussein S.H., Khekan A.R., Rashid M., Al-Msari H., Tran T.H. (2021). Development of ensemble machine learning approaches for designing fiber-reinforced polymer composite strain prediction model. Eng. Comput..

[40] Arafa S., Milad A., Yusoff N.I.M., Al-Ansari N., Yaseen Z.M. (2021). Investigation into the permeability and strength of pervious geopolymer concrete containing coated biomass aggregate material. J. Mater. Res. Technol..

[41] Kheder G.F. (1999). A two stage procedure for assessment of in situ concrete strength using combined non-destructive testing. Mater. Struct..

[42] Mansour M.Y., Dicleli M., Lee J.-Y., Zhang J.J.E.S. (2004). Predicting the shear strength of reinforced concrete beams using artificial neural networks. Eng. Struct..

[43] Naderpour H., Poursaeidi O., Ahmadi M.J.M. (2018). Shear resistance prediction of concrete beams reinforced by FRP bars using artificial neural networks. Measurement.

[44] Ahmad A., Chaiyasarn K., Farooq F., Ahmad W., Suparp S., Aslam F. (2021). Compressive Strength Prediction via Gene Expression Programming (GEP) and Artificial Neural Network (ANN) for Concrete Containing RCA. Buildings.

[45] Song H., Ahmad A., Ostrowski K.A., Dudek M. (2021). Analyzing the Compressive Strength of Ceramic Waste-Based Concrete Using Experiment and Artificial Neural Network (ANN) Approach. Materials.

[46] Nguyen H., Vu T., Vo T.P., Thai H.-T. (2021). Efficient machine learning models for prediction of concrete strengths. Constr. Build. Mater..

[47] Dao D.V., Trinh S.H., Ly H.-B., Pham B.T.J.A.S. (2019). Prediction of compressive strength of geopolymer concrete using entirely steel slag aggregates: Novel hybrid artificial intelligence approaches. Appl. Sci..

[48] Dao D.V., Ly H.-B., Trinh S.H., Le T.-T., Pham B.T.J.M. (2019). Artificial intelligence approaches for prediction of compressive strength of geopolymer concrete. Materials.

[49] Sufian M., Ullah S., Ostrowski K.A., Ahmad A., Zia A., Śliwa-Wieczorek K., Siddiq M., Awan A.A. (2021). An Experimental and Empirical Study on the Use of Waste Marble Powder in Construction Material. Materials.

[50] Ahmad A., Farooq F., Niewiadomski P., Ostrowski K., Akbar A., Aslam F., Alyousef R. (2021). Prediction of compressive strength of fly ash based concrete using individual and ensemble algorithm. Materials.

[51] Song Y.-Y., Ying L.U. (2015). Decision tree methods: Applications for classification and prediction. Shanghai Arch. Psychiatry.

[52] Hillebrand E., Medeiros M.C. (2010). The benefits of bagging for forecast models of realized volatility. Econom. Rev..

[53] Ferreira C. (2001). Gene expression programming: A new adaptive algorithm for solving problems. arXiv.

[54] Gholampour A., Gandomi A.H., Ozbakkaloglu T. (2017). New formulations for mechanical properties of recycled aggregate concrete using gene expression programming. Constr. Build. Mater..

[55] Huang J., Sun Y., Zhang J. (2021). Reduction of computational error by optimizing SVR kernel coefficients to simulate concrete compressive strength through the use of a human learning optimization algorithm. Eng. Comput..

[56] Han Q., Gui C., Xu J., Lacidogna G. (2019). A generalized method to predict the compressive strength of high-performance concrete by improved random forest algorithm. Constr. Build. Mater..

[57] Farooq F., Ahmed W., Akbar A., Aslam F., Alyousef R. (2021). Predictive modeling for sustainable high-performance concrete from industrial wastes: A comparison and optimization of models using ensemble learners. J. Clean. Prod..

[58] Aslam F., Farooq F., Amin M.N., Khan K., Waheed A., Akbar A., Javed M.F., Alyousef R., Alabdulijabbar H. (2020). Applications of gene expression programming for estimating compressive strength of high-strength concrete. Adv. Civ. Eng..

[59] Ahmad A., Ostrowski K.A., Maślak M., Farooq F., Mehmood I., Nafees A. (2021). Comparative Study of Supervised Machine Learning Algorithms for Predicting the Compressive Strength of Concrete at High Temperature. Materials.

[60] Song H., Ahmad A., Farooq F., Ostrowski K.A., Maślak M., Czarnecki S., Aslam F.J.C., Materials B. (2021). Predicting the compressive strength of concrete with fly ash admixture using machine learning algorithms. Constr. Build. Mater..

